# Analysis of Complete Genomes of *Propionibacterium acnes* Reveals a Novel Plasmid and Increased Pseudogenes in an Acne Associated Strain

**DOI:** 10.1155/2013/918320

**Published:** 2013-05-13

**Authors:** Gabriela Kasimatis, Sorel Fitz-Gibbon, Shuta Tomida, Marthew Wong, Huiying Li

**Affiliations:** ^1^Department of Molecular and Medical Pharmacology, Crump Institute for Molecular Imaging, University of California, Los Angeles, CA 90095, USA; ^2^UCLA-DOE Institute for Genomics and Proteomics, Los Angeles, CA 90095, USA

## Abstract

The human skin harbors a diverse community of bacteria, including the Gram-positive, anaerobic bacterium *Propionibacterium acnes*. *P. acnes* has historically been linked to the pathogenesis of acne vulgaris, a common skin disease affecting over 80% of all adolescents in the US. To gain insight into potential *P. acnes* pathogenic mechanisms, we previously sequenced the complete genome of a *P. acnes* strain HL096PA1 that is highly associated with acne. In this study, we compared its genome to the first published complete genome KPA171202. HL096PA1 harbors a linear plasmid, pIMPLE-HL096PA1. This is the first described *P. acnes* plasmid. We also observed a five-fold increase of pseudogenes in HL096PA1, several of which encode proteins in carbohydrate transport and metabolism. In addition, our analysis revealed a few island-like genomic regions that are unique to HL096PA1 and a large genomic inversion spanning the ribosomal operons. Together, these findings offer a basis for understanding *P. acnes* virulent properties, host adaptation mechanisms, and its potential role in acne pathogenesis at the strain level. Furthermore, the plasmid identified in HL096PA1 may potentially provide a new opportunity for *P. acnes* genetic manipulation and targeted therapy against specific disease-associated strains.

## 1. Introduction

Acne vulgaris, commonly called acne, is a disease of the pilosebaceous unit of the skin. It affects over 80% of all adolescents in the US [1] and persists into adulthood in 50% of the cases [2, 3]. While the etiology of the disease is undefined, four pathogenic mechanisms have been proposed: increased sebum production, changes in the follicle, hormone, and the activity of the follicular microflora [4–8]. Antibiotic treatment is one of the main acne therapies targeting the microbes living in the follicle.

*Propionibacterium acnes,* a Gram-positive anaerobic bacterium, has been associated with acne pathogenesis, largely due to the fact that it is commonly isolated from acne lesions [9, 10] and that it can cause inflammation in the host skin. Contrarily, *P. acnes* is accepted as a commensal bacterium and in some cases has been shown to play a protective role against invading pathogenic colonization [11]. Our study of the human skin microbiome associated with acne demonstrated that *P. acnes* dominated the pilosebaceous unit in both healthy individuals and acne patients [12]. However, at the strain level, *P. acnes* distributions were significantly different in the two cohorts, suggesting that different *P. acnes* strains may contribute differently to skin health and disease. Therefore, understanding the genetic differences between acne-associated strains and other strains is essential to understanding the phenotypic differences of the strains and their different roles in acne.

Complete genome sequences provide detailed insights into genetic variations among strains, which may explain their phenotypic differences. We previously sequenced a complete *P. acnes* genome, HL096PA1 [12]. This strain belongs to *recA* type IA and ribotype (RT) 5, which is highly associated with acne. It is resistant to multiple antibiotics, including tetracycline, clindamycin, and erythromycin with resistance-conferring mutations in the 16S ribosomal RNA (rRNA) gene (G1058C) and the 23S rRNA gene (A2058G). To date, HL096PA1 is the only available complete genome of acne-associated strains [12]. The first sequenced *P. acnes* strain with a complete genome is KPA171202 [13]. This strain belongs to *recA* type IB and RT1, which was not specifically associated with acne [12]. The KPA171202 genome is 2.56 M bp long with 60% GC content. 2,333 open reading frames (ORFs) are encoded. To investigate whether genomic variations among *P. acnes* strains can explain their differences in virulent properties, in this study we performed a detailed genome comparison of the genome of HL096PA1 to KPA171202.

## 2. Materials and Methods

### 2.1. *P. acnes* HL096PA1 Genome Sequencing, Assembly and Annotation

HL096PA1 was sequenced using Roche/454 FLX as previously described [12]. The genome was finished by multiple long-range PCRs combined with Sanger sequencing. Genome assembly and annotation were previously described [12]. Extensive manual inspection and editing of the genome annotation were performed. The GenBank accession numbers for HL096PA1 chromosome and plasmid pIMPLE-HL096PA1 are CP003293 and CP003294, respectively.

### 2.2. Genome Comparison

*P. acnes* genome visualization and sequence comparison were performed using the ARTEMIS comparison tool (http://www.sanger.ac.uk/software/ACT/) [14]. Best-BLASTp matches with a cutoff *E*-value of 1*E* − 10 were used to identify HL096PA1 and KPA orthologous proteins.

### 2.3. Identification and Verification of Pseudogenes

Predicted partial or truncated HL096PA1 protein-coding ORFs were aligned to homologs or truncated proteins in the nonredundant protein database to identify pseudogenes. For pseudogene verification, primers flanking the gene regions with frameshifts were designed for suspected HL096PA1 pseudogenes. DNA fragments of 500–1,000 bp were generated using PCR. Sanger sequencing was used to sequence the full length of the amplicons to verify the frameshifts.

### 2.4. Verification of Genomic Inversion

PCR targeting the chromosomal inversion region of HL096PA1 was performed using the primer sets described in Figure 3(b). Each 20 *μ*L-reaction contained 13.3 *μ*L molecular grade H_2_O, 2 *μ*L 10X PCR buffer, 0.6 *μ*L 50 mM MgSO_4_, 0.4 *μ*L 10 mM dNTPs, 0.8 *μ*L primers (final concentration of 0.4 *μ*M), 0.1 *μ*L Platinum *Taq* DNA Polymerase High Fidelity (Invitrogen), and 2 *μ*L 30 ng/uL genomic DNA template. Genomic DNA extracted from HL096PA1 or KPA171202 was amplified. Thermocycling conditions were as the following: initial denaturation step of 10 minutes at 94°C, 35 cycles of 45 seconds at 94°C, 45 seconds at 54°C, and 7 minutes at 72°C and a final elongation step of 20 minutes at 72°C. The primer sequences used are the following: P1 (5′-CGCCACAGCATCACTTAAT-3′), P2 (5′-ACTTGTTACCACAAACCTATTCTT-3′), P3 (5′-CAGACCACTTCTCTAACACACA-3′), P4 (5′-GGCTTACCTCAACAATGTTAAA-3′), P5 (5′-CACGCATTGGAATTACAGAG-3′), and P6 (5′-CGAACCTACTCAGATGGAATTAC-3′).

## 3. Results and Discussion

### 3.1. General Genome Features of *P. acnes* Strain HL096PA1

**Figure 1:**
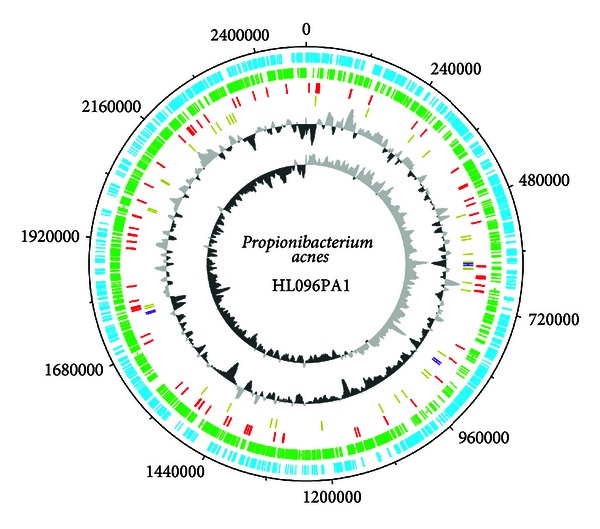
Circular representation of *P. acnes* HL096PA1 chromosome. From the outer circle to the inner circle are circle 1, ORFs on the positive strand (blue); circle 2, ORFs on the negative strand (green); circle 3, pseudogenes (red); circle 4, tRNA (gold) and rRNA (purple); circle 5, GC content (light/dark grey); circle 6, GC skew (light/dark grey). DNA Plotter [46] was used in making the figure.

Similar to other sequenced *P. acnes* strains, HL096PA1 has a circular chromosome of 249, 4191 bp (Figure 1). It encodes three sets of 16S, 23S, and 5S rRNA operons, 45 tRNA genes, and 2,254 protein coding genes. A comparison of the general genome features of HL096PA1 and KAP171202 is shown in Table 1. Although HL096PA1 belongs to a different lineage, it shares 94% of the sequence with the genome of KPA171202. Among the proteins encoded on the HL096PA1 chromosome, 91% are orthologous to KPA171202 proteins (>90% amino acid identity in >60% sequence length). This suggests that small genomic variations or extrachromosomal elements may be responsible for different phenotypes of the two strains and their different associations with acne pathogenesis.

### 3.2. Plasmid in HL096PA1, pIMPLE-HL096PA1

Compared to KPA171202, the most significant specific feature of HL096PA1 is a plasmid. HL096PA1 genome sequencing and assembly revealed a second large contig in addition to the chromosome, which appears to be an extrachromosomal plasmid [12]. The plasmid, named pIMPLE-HL096PA1, is 56,169 bp long. It encodes 74 ORFs (PAGK_2319–PAGK_2392) and one origin of replication (RepA). Several plasmid related proteins, including plasmid partition protein ParA (PAGK_2332) and plasmid stabilization system toxin and antitoxin proteins (PAGK_2321 and PAGK_2322), are encoded. Based on the sequencing coverage and quantitative PCR results, the copy number of the plasmid is three per genome [12]. While our attempts to isolate the plasmid in culture have been unsuccessful possibly due to the low copy number and the large size of the plasmid, we have since identified homologous plasmids in multiple other *P. acnes* strains that belong to the same lineage as HL096PA1 based on genome sequencing [12].

The assembled sequence contig suggests that pIMPLE-HL096PA1 is a linear plasmid, with hairpin ends used for bidirectional replication. Linear plasmids with terminal inverted repeats (TIRs) and 5′ terminally attached proteins have been found in bacteria, fungi, and higher eukaryotes [15–19]. Well-studied examples of human pathogenic bacteria with such linear plasmids include Spirochaete *Borrelia* and Filamentous *Streptomyces* [16, 20–24]. TIRs have been shown to be essential for bidirectional linear plasmid replication [20, 22–24]. Similar to the linear plasmids observed in *Borrelia* and *Streptomyces*, pIMPLE-HL096PA1 contains a 20 bp short TIR with a sequence of ACGACACCAGCACCCACAAC at each end of the plasmid. Due to the difficulty in assembling the repeat sequences at the ends, the lengths of both ends of the plasmid are not fully defined but were assembled to the same. Based on the assembled plasmid sequence, the left arm TIR is located 743 bp from the start of the plasmid sequence, spanning the first ORF (PAGK_2319, an unknown protein). The right arm TIR is located 743 bp from the 3′ end of the plasmid sequence. Analysis of the sequences at the ends of the plasmid reveals potential hairpin structures within the repeat sequences. The last ORF on the plasmid, PAGK_2392, is located near the right arm TIR and encodes resolvase A (ResA). ResA may facilitate fusion and resolution of hairpin telomeres during plasmid replication. It may cut the circular-like plasmid after replication into two linear plasmids with hairpin ends, using a mechanism similar to telomere resolvase (ResT), an enzyme commonly encoded by linear plasmids and shown to facilitate resolution of hairpin telomeres during plasmid replication [25].

The genes encoded on pIMPLE-HL096PA1 have little redundancy with the HL096PA1 chromosome. The plasmid shares only two genes (PAGK_2383 and PAGK_2391) with the chromosome. However, it encodes 12 genes (PAGK_2331, PAGK_2332, and PAGK_2337–PAGK_2346) homologous to the genes in KPA171202, which are absent on the HL096PA1 chromosome and are mostly hypothetical proteins with unknown functions. It also encodes an additional esterase/lipase (PAGK_2386). Lipases have been shown to contribute to growth advantage and possible pathogenicity of *P. acnes* in acne. Lipases hydrolyze triacylglycerols to free fatty acids, which may reduce the invasion of other skin pathogens but may also lead to inflammation and the development of acne when over-produced [26, 27].

**Figure 2:**
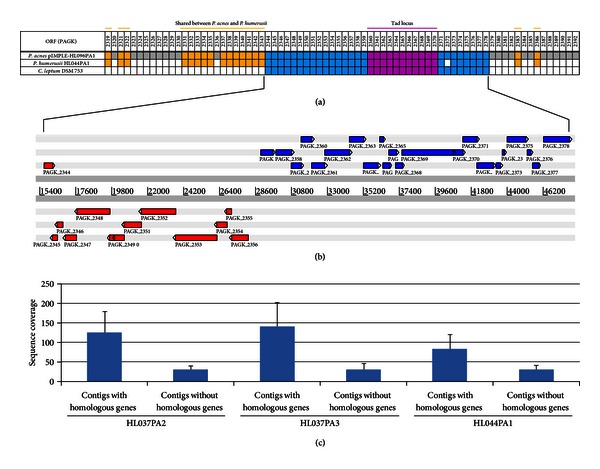
The homologous regions among *P. acnes* pIMPLE-HL096PA1, *P. humerusii* HL044PA1, and *C. leptum* DSM 753. (a) The 74 ORFs encoded on the plasmid pIMPLE-HL096PA1 are shown. ORFs PAGK_2344–PAGK_2378 (blue), including the Tad locus (pink), are shared among all three genomes. In addition, several ORFs are shared between *P. acnes* and *P. humerusii* (orange). ORFs unique to *P. acnes *are shown in grey. Absence of *P. acnes *homologous ORFs in *P. humerusii* or *C. leptum* is shown in white. (b) Organizations of the ORFs shared among all three genomes. (c) The sequence coverage of the contigs in three *P. humerusii* strains (HL037PA2, HL037PA3, and HL044PA1) that share homologous genes with pIMPLE-HL096PA1 is 3–5-folds higher than the contigs that do not share homologous genes with pIMPLE-HL096PA1.

Significant horizontal gene transfer is evident within pIMPLE-HL096PA1. Thirty-five of the 74 predicted ORFs (PAGK_2344–PAGK_2378) are highly homologous to ORFs CLOLEP_00122–CLOLEP_00166 encoded in *Clostridium leptum* DSM 753 (*E*-value = 1*E* − 100 to 0) (Figure 2(a)), a Gram-positive commensal bacterium found in the human gut. The same region is also highly homologous to the genomes of *Propionibacterium humerusii* strains HL037PA2, HL037PA3, HL044PA1, and P08 [28]. *P. humerusii* is a bacterium found on the human skin [12] and is closely related to *P. acnes*. It shares several additional ORFs with pIMPLE-HL096PA1 (Figure 2(a)). Based on our sequencing data of strains HL037PA2, HL037PA3, and HL044PA1, the homologous genes in these *P. humerusii *genomes had a three to five folds higher sequencing coverage than other genes in the genomes (Figure 2(c)). Although these genomes are not complete genomes, the higher sequencing coverage of these genes suggests that they may also be located on an extrachromosomal mobile genetic element in *P. humerusii*. It is unclear whether this set of ORFs was horizontally transferred among *P. acnes, P. humerusii*, and *C. leptum* or transferred from an ancestor organism. Further investigation into the relationships among these microbes that primarily reside at different body sites will provide insight to how genetic materials were transferred and how this mechanism affected the virulent properties of human commensals.

Within this cluster of *C. leptum* homologs on pIMPLE-HL096PA1, we identified a Tad (tight adhesion) locus [12]. Tad locus was reported to mediate colonization, pathogenesis, and tight adhesion of bacteria to host cell surfaces through Flp pilus assembly in Gram-positive pathogens, such as *Corynebacterium dipheriae, Mycobacterium tuberculosis, Mycobacterium bovis, *and* Streptomyces coelicor *[29–31]. The organizations and annotations of the ORFs in this locus are shown in Figure 2 and Table 2. ORFs PAGK_2360–PAGK_2370 encode a set of Tad proteins, including Flp (fimbrial low molecular-weight proteins) pilus proteins, TadZ, TadA, TadB, TadC, and TadE. The transcription of Tad locus in HL096PA1 appears to be controlled by a sigma 70 family transcription factor (PAGK_2357) and an antisigma factor (PAGK_2358) located immediately upstream of the Tad locus. Sigma 70 family transcription factors are commonly found upstream of virulence-associated gene loci and have been shown to regulate virulence expression in response to particular stress-related stimuli in several pathogenic bacteria such as *Vibrio cholera* [32]. This suggests that Tad locus expression in HL096PA1 may be induced under specific conditions, leading to enhanced colonization and adhesion to host cells. While KPA171202 does not possess such a plasmid and the Tad locus, majority of the RT4 and RT5 strains of *P. acnes*, which are highly associated with acne, contain a homologous plasmid and the Tad locus [12]. The unique presence of such a plasmid in acne associated strains may potentially explain their role in acne pathogenesis and can possibly be used as a pathogenicity marker.

### 3.3. Pseudogenes in HL096PA1

Pseudogenes tend to be abundant in bacterial species that have recently adapted to eukaryotic hosts or a pathogenic lifestyle [33]. Thus identification of pseudogenes in disease associated strains may provide insight to their host adaptation and pathogenic mechanisms. We identified 79 predicted pseudogenes in HL096PA1 genome (Table 3). In addition to confirmation of the pseudogenes based on the high-quality 454 sequence reads, we selected 17 out of the 79 identified pseudogenes and confirmed the frameshifts in all 17 genes by PCR and Sanger sequencing. Most pseudogenes represent recent gene disruptions and are usually unique to a particular genome [34]. HL096PA1 and KPA171202 share only four predicted pseudogenes. Compared to the 14 frameshifted ORFs encoded in the KPA171202 genome [13], HL096PA1 harbors a significantly increased number of pseudogenes. This may suggest a faster reduction rate of the chromosome of this strain. Sixty-one of the 79 predicted pseudogenes in HL096PA1 contain single inactivating mutations, indicating that the majority of these mutations were recently acquired [34].

Although the functions of the predicted pseudogenes in HL096PA1 are diverse, 14 of the 79 pseudogenes belong to the functional group of carbohydrate transport and metabolism, including five phosphotransferase system (PTS) components responsible for carbohydrate regulation and sensing. Two of the three predicted fructose PTSs contain frameshifts, suggesting that only one fructose uptake system remains functional in the genome [35]. In addition, HL096PA1 contains two pseudogenes in the only predicted galactitol PTS [35] and one disrupted component in the single glucitol PTS. This suggests that both of these systems are nonfunctional in this strain. These gene disruptions observed in the PTS components and other proteins of the carbohydrate transport and metabolism pathways may reflect an adaptation of HL096PA1 to the availability of nutrients in the pilosebaceous unit, where the major carbon source is lipids but not carbohydrates.

In addition to PTS genes, multiple virulence-associated adhesion proteins in HL096PA1 harbor frameshifts. Adhesion proteins are of special interest because of their pathogenic potential and involvement in biofilm formation. Brüggemann et al. reported nine putative adhesion proteins in KPA171202 genome which contain thrombospondin type 3 repeats, PKD and SEST domains [13]. We found frameshifts in five of the nine orthologous adhesion proteins in HL096PA1, including PAGK_1592, PAGK_1593, PAGK_1821, PAGK_1897, and PAGK_2110. These orthologs were also recently reported to contain frameshifts in *P. acnes* strain 266 [36], which is a strain more closely related to HL096PA1 than to KPA171202. The differences in functional adhesion protein genes among the strains suggest that various *P. acnes* strains may use antigenic variations, a mechanism used by many pathogenic bacteria to avoid the host immune system [37], as a host immune invasion mechanism.

### 3.4. Genomic Islands in HL096PA1

HL096PA1 harbors several island-like genomic regions. In KPA171202, 11 genomic islands were reported previously [13]. These regions encode proteins associated with pathogenicity or survival, mobility, and proteins of unknown function. The chromosome of HL096PA1 shares eight of the island-like regions found in KPA171202. Three of the KPA171202 island-like regions are absent in HL096PA1, including PPA839-878, PPA1278-1321, and PPA1576-1618. In addition to the genomic islands described by Brüggemann et al. [13], an island-like region (PPA2055-2092) in KPA171202 reported later [36] is also absent in HL096PA1. On the other hand, HL096PA1 contains two unique genomic islands, locus 1 and locus 2, which have been shown to be associated with the disease state of acne [12]. These two loci are absent in KPA171202 but present in all strains that belong to the same lineage as HL096 PA1 [12], including almost all RT4 and RT5 strains and another completely sequenced strain SK137 [36].

HL096PA1 locus 1 spans over 6 Kb and encodes seven ORFs (PAGK_0121–PAGK_0127). This region shows evidence of a cryptic phage-like integration through the flanking tRNAs. It encodes phage-related genes such as site-specific recombinase. An antibiotic ABC transporter is also encoded in this region; however, it is a predicted pseudogene, suggesting that antibiotic resistance was once conferred through this phage integration.

HL096PA1 locus 2 spans over 20 Kb and encodes 23 ORFs (PAGK_0160–PAGK_0182). Within this genomic island, a complete streptolysin S (SLS) biosynthesis gene cluster (PAGK_0168–PAGK_0174) is encoded, including CAAX amino protease family protein (PAGK_0170), streptolysin prototoxin protein SagA (PAGK_0171), cyclodehydratase genes SagD, SagC, and SagB (PAGK_0172–PAGK_0174), and two hypothetical proteins (PAGK_0168 and PAGK_0169). Streptolysin biosynthetic cluster is commonly found in Gram-positive mammalian pathogens such as *Clostridium botulinum, Listeria monocytogenes,* and *Staphylococcus aureus* [38]. This gene cluster is potentially capable of producing a nonribosomally synthesized secreted toxin, streptolysin. Streptolysin is responsible for the classical beta-hemolytic phenotype of bacterial colonies grown on blood agar media. This gene cluster in HL096PA1 may contribute to its virulence against the host and to its dominant colonization against other pilosebaceous unit dwelling microbes.

The second major group of genes encoded in locus 2 is phage defense genes. By using Pfam [39], we identified an abortive infection Abi_2 domain (pfam07751) within ORF PAGK_0180, which is involved in bacteriophage resistance via premature cell death upon phage entry. This abortive infection defense mechanism against bacterial phage is mediated by a single copy of Abi_2 domain containing protein [40]. A second copy of this Abi_2 domain containing gene, PAGK_2391, is encoded on the plasmid pIMPLE-HL096PA1. This suggests a possible common origin of locus 2 and the plasmid.

Additional genomic evidence supports a possible common origin of locus 2 and the plasmid pIMPLE-HL096PA1. Located at the immediate downstream of the abortive infection defense system is ORF PAGK_0182. It is a hypothetical protein homologous to CLOLEP_00167 in *C. leptum*. As described above, the plasmid pIMPLE-HL096PA1 contains a cluster of genes with high similarities to *C. leptum* DSM753 genes CLOLEP_00122–CLOLEP_00166, while the homolog of CLOLEP_00167 lies in locus 2 on the chromosome of HL096PA1. This further suggests a possible common origin of locus 2 and the plasmid. Notably, this gene in locus 2, PAGK_0182, contains a 31 bp sequence. As reported by Fitz-Gibbon et al. [12], this 31 bp sequence was found identical to the clustered regularly interspaced short palindromic repeat (CRISPR) spacer sequence encoded in some of the type II *P. acnes* strains. This suggests that the genes in locus 2 may have been acquired through a phage or plasmid mechanism in RT4 and RT5 strains, while type II strains do not harbor this genomic island possibly due to the protection mechanism of CRISPR/cas system against foreign DNA.

### 3.5. Genomic Inversion in HL096PA1

**Figure 3:**
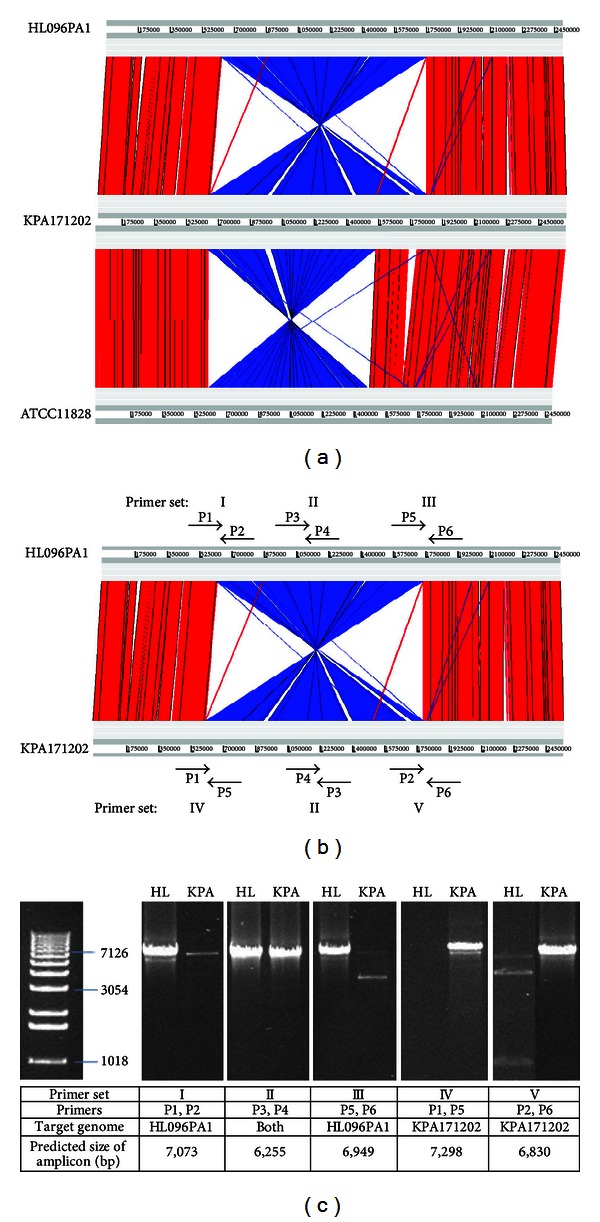
Genomic inversion in HL096PA1. (a) Genomic inversions observed in *P. acnes* strains HL096PA1 and ATCC11828 compared to *P. acnes* strain KPA171202. (b) Primer sets I–V designed to verify the genomic inversion in HL096PA1. (c) PCR amplified DNA fragments of the inverted regions shown on an agarose gel. Primers, target genomes, and the predicted sizes of the amplicons are shown in the table. HL, strain HL096PA1; KPA, strain KPA171202.

While genomic inversion is commonly seen in bacterial genomes, pathogenic bacteria often exhibit a high degree of genomic rearrangement [41]. We observed a large genomic inversion in HL096PA1 when compared to KPA171202. The inversion spans the ribosomal region of 1.2 Mb ranging from the 1st set to the 3rd set of ribosomal operons (Figure 3(a)). To verify the inversion, we designed primer sets that span the beginning, middle, and the end of the inverted region (Figure 3(b)). PCR amplifications of these regions were performed. As predicted based on the inverted genomic region, HL096PA1 had strong amplifications of the regions covered by primer sets I, II, and III but had no specific amplifications using primer sets IV and V (Figure 3(c)). The inversion was further confirmed by sequencing the ends of the amplicons using the Sanger method. KPA171202 was used as a control. As predicted, KPA171202 had no specific amplifications using primer sets I and III, but strong amplifications using primer sets II, IV, and V. Among the nine currently available complete *P. acnes* genomes, ATCC11828 strain [42] also contains a genomic inversion. Similar to the inversion in HL096PA1, it spans a ribosomal region of 0.9 Mb including the 1st set and the 2nd set of ribosomal operons (Figure 3(a)).

## 4. Conclusions

Our recent study of the skin microbiome has shown that *P. acnes* resides in the pilosebaceous unit of both healthy skin and acne affected skin [12], challenging the traditional concept applied in clinical practice that all *P. acnes* contributes to acne pathogenesis. Moreover, several studies of the *P. acnes* strain populations on the skin suggested that acne-associated *P. acnes* subpopulations exist and may correlate with the genomic variations of the strains [12, 22, 36, 43, 44]. Our genome sequencing and comparison of a large number of strains revealed significant disease- or health-associated genetic elements [12]; however, detailed analyses of extrachromosomal elements, pseudogenes, and genome structure variations were limited due to unfinished genome sequences. The comparison between two complete genomes HL096PA1 and KPA171202 described in this study allowed us to better understand the strain-specific genomic variations in plasmid, pseudogenes, genomic islands, and genomic inversion that may be associated with disease pathogenesis.

In this comparison, we identified several strain-specific genetic elements that are potentially associated with increased virulence of HL096PA1 in acne. Present in HL096PA1 and absent in KPA171202 is the first described *P. acnes* plasmid carrying a tight adhesion locus, which has been shown to enhance colonization and biofilm formation in several human pathogens [29–31]. Further investigation of this *P. acnes* plasmid will provide insight on its pathogenic potential. Compared to KPA171202, we observed a more than five-fold increase in the number of pseudogenes in HL096PA1, which suggests a potential mechanism of this strain to host adaptation. HL096PA1 contains two genomic islands that are unique to acne-associated strains and may be associated with increased virulence of this lineage in acne [12]. In addition, a large genomic inversion was observed in HL096PA1. Together these genetic differences revealed by complete genome comparison provide insights into the complexity of the strain variations of this organism and support the existence of specific *P. acnes* subpopulations which may be associated with acne pathogenesis [36, 45]. Furthermore, the first *P. acnes* plasmid identified in HL096PA1 may potentially provide a new opportunity for genetic manipulation of this organism. The knowledge gained from this comparative study may potentially lead to novel diagnostic and targeted therapeutic approaches for acne against a specific disease associated lineage of *P. acnes*.

## Figures and Tables

**Table 1:** Comparison of the general features of HL096PA1 and KPA171202 genomes.

Features	HL096PA1		KPA171202
	Chromosome	Plasmid	Chromosome
*recA* phylotype	IA		IB
Ribotype (RT)	5		1
Acne association	Yes		No
Size (bp)	2,494,191	56,169	2,560,265
GC content (%)	60	62	60
Coding sequences	2,254	74	2,297
Pseudogenes	79	0	14
Coding density (%)	90	83	91
tRNAs	45	0	45
RNA operons	3	0	3
Sequencing method	454/Roche	454/Roche	Sanger
Sequencing coverage	50X		8.7X

**Table 2:** Annotations of the Tad locus in pIMPLE-HL096PA1.

ORF	Tad homolog	Function	Pfam ID	Pfam *E*-value
PAGK_2360	RcpC	Flp pilus assembly protein, rough colony protein-C	SAF	9.60*E* − 05
PAGK_2361	TadZ/A	ATPase	N/A	N/A
PAGK_2362	TadA	ATPase	GSPII_E	1.50*E* − 10
PAGK_2363	TadB/C	Flp pilus assembly protein	GSPII_F	2.10*E* − 05
PAGK_2364	TadB/C	Flp pilus assembly protein	GSPII_F	0.014
PAGK_2365	N/A	Unknown	N/A	N/A
PAGK_2366	TadE	Minor Flp pilus assembly protein	TadE	0.0015
PAGK_2367	TadE	Minor Flp pilus assembly protein	TadE	0.018
PAGK_2368	TadE	Minor Flp pilus assembly protein	TadE	3.00*E* − 07
PAGK_2369	TadB	Flp pilus assembly protein	LysM, BTAD	3.10*E* − 10
PAGK_2370	RcpB	Flp assembly protein, rough colony protein-B	Pilus, CpaD	0.53

**Table 3:** Annotations of the 79 predicted pseudogenes in HL096PA1.

Number	ORF	Start	End	Function	Note
1	PAGK_0006	7,621	6,061	ABC transporter, ATP-binding protein	Shared with KPA171202
2	PAGK_0019	23,673	23,196	PTS system, galactitol-specific IIA component	Confirmed by PCR and Sanger sequencing
3	PAGK_0020	24,985	23,730	PTS system, galactitol-specific IIC component	Confirmed by PCR and Sanger sequencing
4	PAGK_0024	25,937	27,844	Hypothetical protein	Shared with KPA171202
5	PAGK_0025	28,692	28,418	Hypothetical protein	
6	PAGK_0026	29,964	29,294	Hypothetical protein	
7	PAGK_0089	100,317	102,505	Glycosyl hydrolase family protein	
8	PAGK_0123	149,702	148,105	Site-specific recombinase	
9	PAGK_0193	226,656	225,834	Regulator protein	
10	PAGK_0224	266,991	265,055	Putative RHS-related protein	
11	PAGK_0243	289,824	290,724	Putative ABC transporter	
12	PAGK_0352	402,744	401,670	Hypothetical protein	Confirmed by PCR and Sanger sequencing
13	PAGK_0393	441,551	441,884	2-Hydroxy acid dehydrogenase	
14	PAGK_0395	444,633	446,378	Hypothetical protein	
15	PAGK_0429	474,851	476,017	Putative sensor histidine kinase, two component system	
16	PAGK_0440	487,718	487,064	Hypothetical protein	
17	PAGK_0463	510,587	509,659	Phosphotransferase system protein mannitol/fructose-specific IIA subunit	Confirmed by PCR and Sanger sequencing
18	PAGK_0482	529,279	530,146	Myo-inositol catabolism IolH protein	
19	PAGK_0573	630,324	628,497	Hypothetical protein	
20	PAGK_0589	653,210	651,537	Alpha-glucosidase/amylase family protein	
21	PAGK_0592	657,824	655,229	Aminopeptidase N	
22	PAGK_0601	674,112	671,871	UvrD/Rep helicase	
23	PAGK_0603	685,081	680,455	Hypothetical protein	
24	PAGK_0604	687,943	685,068	Putative helicase	
25	PAGK_0613	702,394	698,431	Hypothetical protein	
26	PAGK_0720	820,262	819,153	Putative peptide ABC transporter, permease component	
27	PAGK_0757	855,259	854,123	Putative lysophospholipase	Confirmed by PCR and Sanger sequencing
28	PAGK_0811	908,541	907,748	Hypothetical protein	
29	PAGK_0875	986,883	986,286	Hypothetical protein	
30	PAGK_0944	1,060,628	1,059,358	Prephenate dehydrogenase	
31	PAGK_1004	1,127,197	1,126,813	Hypothetical protein	
32	PAGK_1011	1,133,493	1,133,051	Hypothetical protein	
33	PAGK_1158	1,298,563	1,297,552	Putative oxidoreductase protein	Confirmed by PCR and Sanger sequencing
34	PAGK_1160	1,299,462	1,299,708	Beta-glucosidase fragment	Confirmed by PCR and Sanger sequencing
35	PAGK_1181	1,318,788	1,319,584	Putative hydrolase	Confirmed by PCR and Sanger sequencing
36	PAGK_1233	1,374,383	1,376,290	PTS system, mannitol-specific IIABC component	Confirmed by PCR and Sanger sequencing
37	PAGK_1238	1,380,347	1,381,136	Putative metallo-beta-lactamase	
38	PAGK_1242	1,386,373	1,384,720	Acetyl-coenzyme A synthetase	
39	PAGK_1275	1,426,760	1,425,953	GntR family regulatory protein	
40	PAGK_1276	1,426,762	1,427,445	Hypothetical protein	
41	PAGK_1284	1,434,222	1,434,422	Hypothetical protein	
42	PAGK_1285	1,434,707	1,435,190	Transposase, Mutator family	
43	PAGK_1332	1,481,350	1,482,114	Hypothetical protein	Confirmed by PCR and Sanger sequencing
44	PAGK_1344	1,496,744	1,495,088	Hypothetical protein	
45	PAGK_1347	1,501,212	1,499,580	ABC transporter ATP-binding protein	Confirmed by PCR and Sanger sequencing
46	PAGK_1354	1,508,597	1,511,439	Oxidoreductase, putative D-lactate	
47	PAGK_1387	1,549,654	1,548,834	Putative GntR-family transcriptional regulator	
48	PAGK_1407	1,567,445	1,569,345	Hypothetical protein	
49	PAGK_1536	1,703,687	1,704,612	Putative lysophospholipase	
50	PAGK_1586	1,764,348	1,765,387	Binding-protein-dependent transport system inner membrane component	Confirmed by PCR and Sanger sequencing and shared with *P. acnes* J139
51	PAGK_1587	1,765,384	1,766,265	Binding-protein-dependent transport system inner membrane component	Shared with *P. acnes* J139
52	PAGK_1592	1,770,327	1,769,760	Protein associated to putative adhesion protein	
53	PAGK_1593	1,771,040	1,770,487	Protein associated to putative adhesion protein	
54	PAGK_1594	1,773,223	1,771,037	Hypothetical protein	
55	PAGK_1608	1,786,154	1,785,310	Hypothetical protein	
56	PAGK_1643	1,820,268	1,818,959	Transporter (sodium : sulfate symporter)	Shared with KPA171202
57	PAGK_1717	1,907,494	1,905,155	Putative ABC transporter-associated permease	Confirmed by PCR and Sanger sequencing
58	PAGK_1730	1,924,158	1,920,911	Endo-beta-N-acetylglucosaminidase family protein	
59	PAGK_1742	1,935,480	1,935,974	Hypothetical protein	
60	PAGK_1744	1,936,979	1,938,333	Putative sialidase	
61	PAGK_1760	1,952,431	1,951,577	Glycerophosphoryl diester phosphodiesterase	
62	PAGK_1793	1,981,847	1,978,587	Hypothetical protein	Confirmed by PCR and Sanger sequencing
63	PAGK_1821	2,014,629	2,016,701	Hypothetical protein	Confirmed by PCR and Sanger sequencing
64	PAGK_1858	2,054,370	2,056,156	ABC transporter ATP-binding protein	
65	PAGK_1895	2,103,920	2,105,327	Pyridine nucleotide-disulphide oxidoreductase	
66	PAGK_1897	2,107,483	2,109,669	Hypothetical protein	Shared with *P. acnes* J139
67	PAGK_1966	2,185,427	2,184,028	Amidase	
68	PAGK_1990	2,207,618	2,209,728	5′-Nucleotidase/2′,3′-cyclic phosphodiesterase	
69	PAGK_1995	2,211,776	2,211,311	Hypothetical protein	
70	PAGK_1997	2,212,029	2,212,392	Hypothetical protein	
71	PAGK_2003	2,218,626	2,218,090	Hypothetical protein	
72	PAGK_2008	2,222,549	2,223,462	Putative asparaginase	
73	PAGK_2023	2,236,080	2,235,748	Ornithine carbamoyltransferase, catabolic	
74	PAGK_2041	2,255,990	2,257,066	Putative lysophospholipase	
75	PAGK_2110	2,327,052	2,328,480	Putative adhesion or S-layer protein	Confirmed by PCR and Sanger sequencing
76	PAGK_2118	2,338,399	2,337,074	Hypothetical protein	
77	PAGK_2159	2,375,435	2,374,944	MarR family transcriptional regulator	Confirmed by PCR and Sanger sequencing
78	PAGK_2189	2,405,633	2,407,955	D-ornithine aminomutase E component	
79	PAGK_2225	2,450,551	2,449,750	SorC family transcriptional regulator	Shared with KPA171202
